# Metabolic Changes in Serum Metabolome of Beagle Dogs Fed Black Ginseng

**DOI:** 10.3390/metabo10120517

**Published:** 2020-12-19

**Authors:** Dahye Yoon, Ye Jin Kim, Wan Kyu Lee, Bo Ram Choi, Seon Min Oh, Young Seob Lee, Jae Kwang Kim, Dae Young Lee

**Affiliations:** 1 Department of Herbal Crop Research, National Institute of Horticultural and Herbal Science, RDA, Eumseong 27709, Korea; dahyeyoon@korea.kr (D.Y.); bmcbr@korea.kr (B.R.C.); seonmin88@korea.kr (S.M.O.); youngseoblee@korea.kr (Y.S.L.); 2 Division of Life Sciences, College of Life Sciences and Bioengineering, Incheon National University, Yeonsugu, Incheon 22012, Korea; 201721047@inu.ac.kr (Y.J.K.); kjkpj@inu.ac.kr (J.K.K.); 3 College of Veterinary Medicine, Chungbuk National University, Cheongju 28644, Korea; wklee@chungbuk.ac.kr

**Keywords:** black ginseng, *Panax ginseng*, HR-MAS NMR, metabolomics, dog

## Abstract

The effects of black ginseng, which has many kinds of biological activities, on dogs was investigated. Serum samples of beagle dogs, which were fed with black ginseng for 8 weeks, were measured using high-resolution magic angle spinning (HR-MAS) nuclear magnetic resonance (NMR) spectrometry. Acquired NMR data from the serum of dogs fed for 0, 4, and 8 weeks were analyzed by metabolic profiling and multivariate statistical analysis. In statistical analysis and biomarker analysis results of metabolite profiles, formate, glutamine, histidine, isoleucine, leucine, proline, and valine had variable importance in projection (VIP) scores above 1.0 and excellent area under the curve (AUC) values of receiver operating characteristic (ROC) curves above 0.9. In the result of multivariate statistical analysis, the score plot showed the discrimination between before and after feeding of black ginseng. These differences in metabolic profiles are considered to be due to the involvement of metabolic processes following black ginseng administration, such as enhancing immunity and energy metabolism. Through metabolomics analysis, we confirmed the biological efficacy of black ginseng in dogs and also confirmed that metabolomics can be applied to the pet health industry.

## 1. Introduction

Ginseng has been used as a natural medicinal ingredient in East Asia [1]. *Panax ginseng* C.A. Meyer is processed to diversify efficacy. Ginsenosides, known to have biological functions, differ in composition depending on the processes of raw ginseng used [2]. Black ginseng is prepared from white ginseng by repeated steaming and drying for nine cycles [3]. A previous study reported that black ginseng contains more ginsenoside Rd, Rg3, Rk1, and Rg5 than other processed ginseng products [2]. Studies on biological activities of black ginseng such as anticancer effects [3,4,5,6,7], antioxidant activities [8,9,10,11], ameliorative effects on obesity, hyperglycemia, diabetes related to metabolic syndrome [12,13,14,15,16,17,18], and effects of antiwrinkle and skin-whitening related to cosmetics [19,20], have been conducted.

Increased interest in healthcare has led to the emergence of the functional food market. These foods are not only for people but for the pet market as well, resulting in a new trend of healthcare for pets. Especially from the viewpoint of veterinary care, metabolomics research is increasing [21]. Metabolomics is the study of the overall changes in metabolites, the end products of metabolism. Metabolites represent the product of actual reaction as well as step-by-step products. Various tools such as nuclear magnetic resonance (NMR) spectroscopy and mass spectrometry (MS) can be used in metabolomics. Metabolic approaches to dog studies such as those on species [22,23], diets [24,25], and diseases [26,27,28] have been conducted. MS instruments were frequently used for these metabolomics studies, and MS instruments attached to gas-chromatography (GC) and liquid-chromatography (LC) equipment were used to measure plasma and serum samples in studies of metabolic changes arising from diets [24,25]. A previous study has reported that metabolic changes according to feeding of two concentrations of black ginseng on dogs were analyzed by GC-TOF/MS, and the research confirmed the potential anti-inflammatory effect [29]. In this study, high-resolution magic angle spinning (HR-MAS) NMR spectroscopy was used for metabolomics study. NMR spectroscopy requires little pretreatment and provides high reproducibility [30]. HR-MAS NMR spectroscopy requires simple sample preparation and a lower amount of sample than liquid NMR spectroscopy. If the amount is sufficient, serum samples are usually analyzed using liquid NMR spectroscopy. However, in this experiment, a small amount of sample could be used for an NMR experiment due to other serum experiments. Since a serum volume of about 50 μL is sufficient for a HR-MAS NMR experiment, it was possible to minimize the amount used for NMR experiments. When measuring semisolid samples, such as blood with high viscosity, broad signals are obtained from the NMR spectrometer by anisotropic interactions. However, these interactions can vanish when the sample is rotated to a magic angle (54.74°), and then liquid-like spectra can be obtained [31].

In this study, dog serum was measured using HR-MAS NMR spectroscopy to confirm changes of metabolites caused by black ginseng. With the use of black ginseng in an animal model, a metabolomics study can not only detect altered metabolites but can also explain the efficacy of black ginseng. This study aimed to explore the effects on pet health of feeding dogs with black ginseng.

## 2. Results

**Figure 1:**
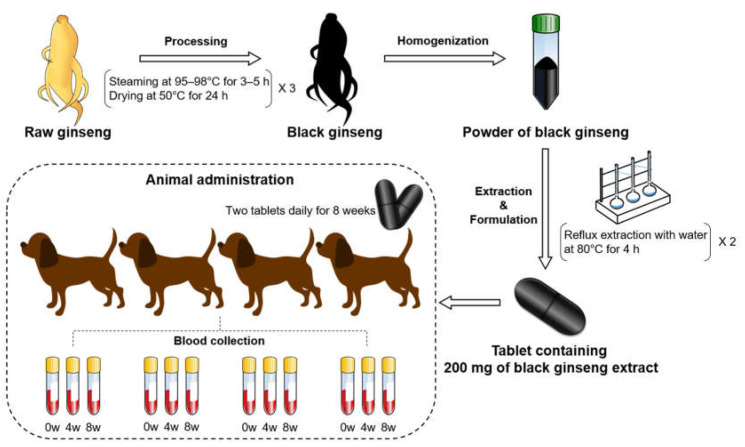
Scheme of this study from the processing of raw ginseng to animal administration of black ginseng.

In this study, black ginseng, which was processed from raw ginseng, was extracted and formulated as a tablet. Tablets containing 200 mg of black ginseng were administrated to the dogs. Their blood was then collected for the metabolomics study. Figure 1 shows the overall scheme of this study. Dog serum samples were analyzed by NMR spectrometry to profile metabolites in order to compare the differences after the feeding of black ginseng.

**Figure 2:**
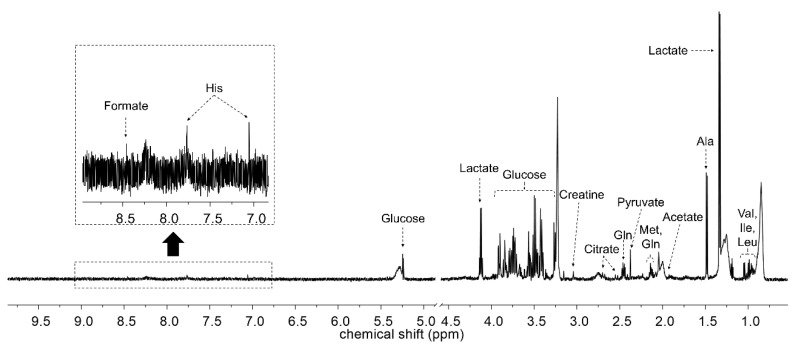
Representative 600 MHz high-resolution magic angle spinning (HR-MAS) nuclear magnetic resonance (NMR) spectrum of dog serum. Residual water area was excluded.

Figure 2 shows the representative HR-MAS NMR spectrum of dog serum. The major metabolites are annotated on the spectrum. These metabolites, including amino acids (valine, isoleucine, leucine, alanine, methionine, glutamine, and glutamate), organic acids (lactate, acetate, pyruvate, citrate, and formate), and glucose, were identified and quantified (Table 1).

Glucose showed the highest contents in the serum samples, and the amount of glucose showed a time-dependent increase. Isoleucine, leucine, and valine, classified as branched chain amino acids (BCAAs), increased after feeding of black ginseng. Among them, valine showed a statistically significant and time-dependent increase. Lactate was the second-highest metabolite in the serum samples, showing a time-dependent decrease, as opposed to glucose. Alanine, glutamine, and histidine significantly increased after feeding of black ginseng. Formate showed a time-dependent and significant decrease.

**Figure 3:**
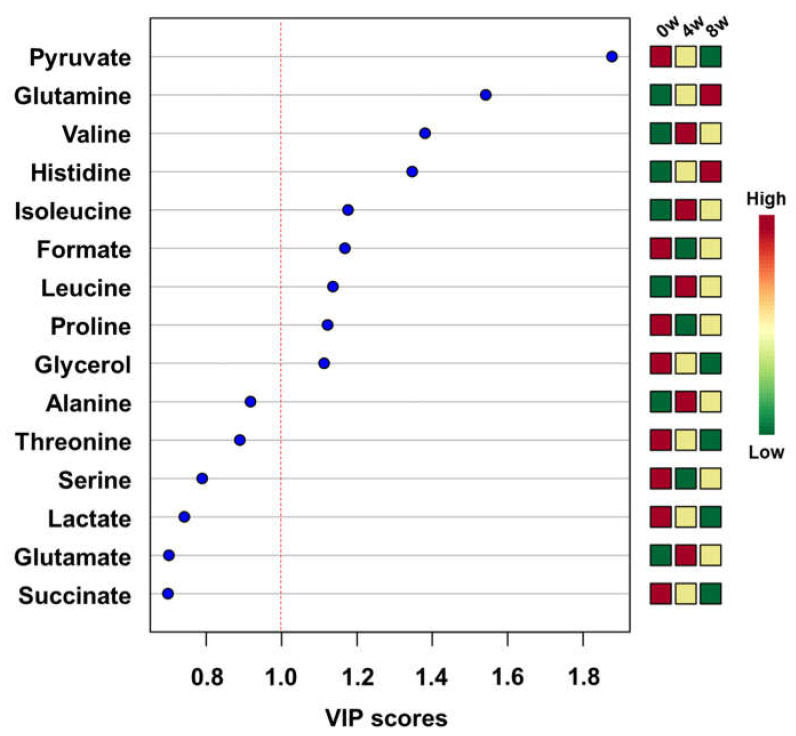
Top 15 metabolites in descending order according to variable importance in projection (VIP) values in the partial least squares discriminant analysis (PLS-DA).

The concentrations of metabolites were statistically analyzed to give the scores of importance for contribution to changes after feeding of black ginseng. The top 15 metabolites are shown in the variable importance in projection (VIP) score plot in descending order (Figure 3). Generally, the metabolites with VIP scores greater than 1.0 importantly contribute to the model [32]. Pyruvate, glutamine, valine, histidine, isoleucine, formate, leucine, proline, and glycerol had VIP scores higher than 1.0. These metabolites were also included in meaningful results of biomarker analysis. The groups were divided before and after feeding of black ginseng for biomarker analysis. Area under the curve (AUC) values were calculated from receiver operating characteristic (ROC) curves. The ROC curve is generally used to confirm the predicted value, but in this study it was used to identify metabolites that showed significant changes after the feeding of black ginseng. An AUC value below 0.7 is considered poor, 0.7–0.8 is moderate, 0.8–0.9 is good, and 0.9–1.0 is excellent [33]. In these results, valine, formate, glutamine, histidine, isoleucine, leucine, and proline showed excellent AUC values; serine, alanine, and glutamate had good AUC values; and glycine, glycerol, pyruvate, methionine, and glucose had moderate AUC values (Table 2).

**Figure 4:**
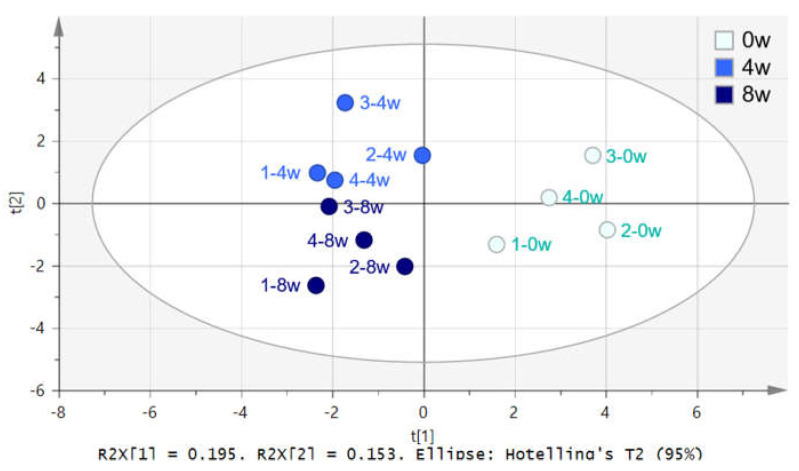
PLS-DA score plot of dog serum 0 (●), 4 (●), and 8 (●) weeks after feeding of black ginseng.

The NMR spectra of dog serum samples were statistically compared using SIMCA software with principal component analysis (PCA) and partial least squares discriminant analysis (PLS-DA). The distribution of all samples was observed using PCA analysis, and the outlier sample was found through the significance level of 0.05. The PLS-DA score plot shows the discrimination between before and after feeding of black ginseng, with 4 and 8 weeks after feeding clustered separately (Figure 4).

## 3. Discussion

The metabolites in the serum were analyzed using NMR spectroscopy with a HR-MAS probe. In general liquid NMR spectroscopy, a sufficient amount of serum is required for the sampling procedure. However, 36 μL of serum was used in this study with HR-MAS NMR spectroscopy. A resolution similar to the liquid NMR spectrum can be obtained with the magic-angle-spinning technique [34]. Moreover, if only a small amount of sample can be obtained from an animal experiment, the HR-MAS technique is advantageous. In this study, the quantified metabolites were statistically compared using univariate and multivariate statistical analyses.

**Figure 5:**
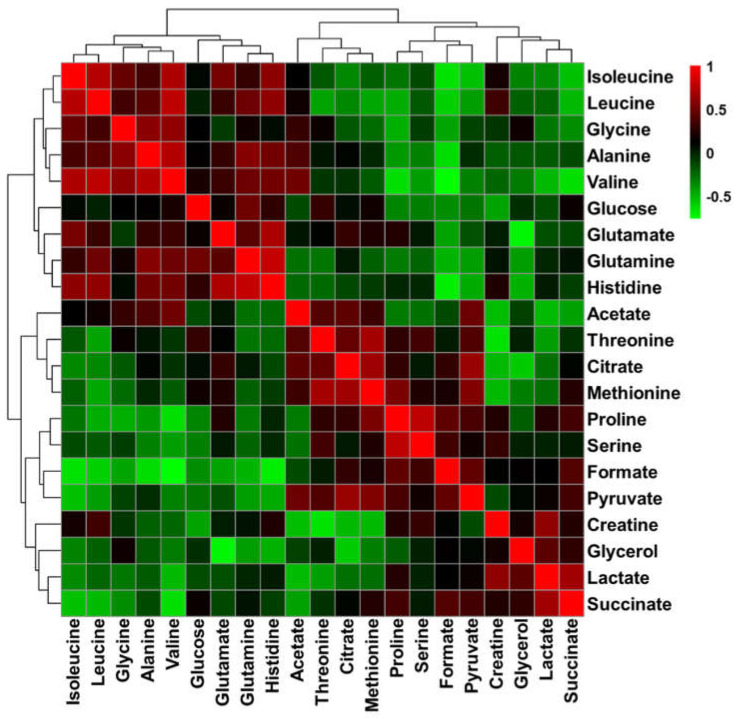
Correlation matrix of metabolites in the serum of dogs. The scale bar shows the correlation coefficient from − 1 to + 1.

Correlation analysis was conducted to sort the metabolites that were statistically correlated (Figure 5). Isoleucine, leucine, glycine, alanine, and valine, which increased after feeding of black ginseng, had a positive correlation. BCAAs, which increased after feeding of black ginseng, have been reported to have a relationship with immune function. BCAAs participate in growth and proliferation of lymphocytes [35]. Jose and Good reported that deficiency of BCAAs impairs immune function in tumor-bearing mice [36]. Tsukishiro and co-workers reported that administration of BCAAs stimulates the local immune system in rats [37]. The administration of black ginseng helped improve immunity by raising the concentration of BCAAs in the dog’s blood. Moreover, the anti-inflammatory response of black ginseng has already been demonstrated in many studies using inflammatory rat models [38,39,40].

**Figure 6:**
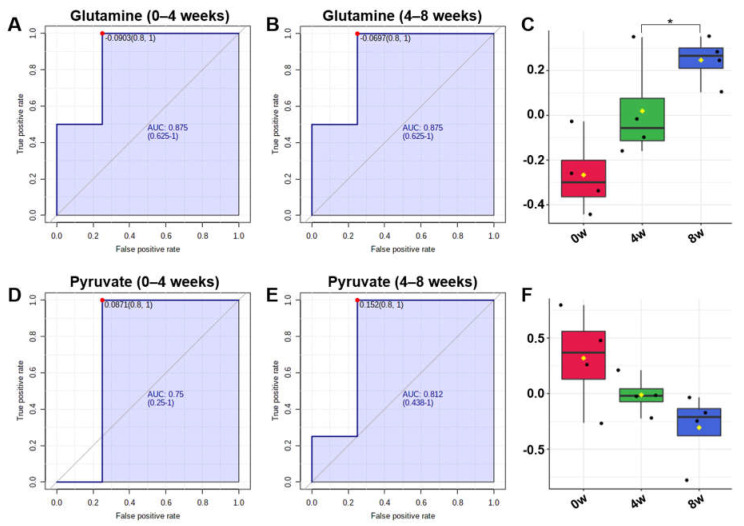
Biomarker analysis of 0 and 4 weeks and 4 and 8 weeks after feeding of black ginseng. (**A**) Receiver operating characteristic (ROC) curves of glutamine comparing 0 and 4 weeks and (**B**) 4 and 8 weeks. (**C**) Box plot of glutamine concentrations (* *p*-value < 0.05). (**D**) ROC curves of pyruvate comparing 0 and 4 weeks and (**E**) 4 and 8 weeks. (**F**) Box plot of pyruvate concentrations.

Metabolites forming a characteristic correlation were glutamate, glutamine, and histidine, with a positive correlation. Histidine is an essential amino acid in adult dogs and cannot be synthesized in the body, and the amount of histidine in the body is increased by ingestion [41]. It can be seen that the amount of histidine increased with the administration of black ginseng. Glutamine formed from histidine is easily interchangeable with glutamate, and in these results, glutamate and glutamine increased with the increase of histidine. In the results of biomarker analysis, glutamine showed a good prediction value of 0.875 in AUC value when comparing 0 and 4 weeks and 4 and 8 weeks after feeding of black ginseng (Figure 6A–C). Pyruvate was one of the metabolites that changed significantly in the results of biomarker analysis (Figure 6D–F). Pyruvate, which is used in energy metabolism, was decreased after feeding of black ginseng. It was reported that polysaccharides in *P. ginseng* promote energy metabolism by increasing the ATP/ADP and ATP/AMP ratios in liver cells [42].

It was seen that blood metabolite concentration was changed by the biological effect of black ginseng or by metabolite conversion as a result of its ingestion.

## 4. Materials and Methods

### 4.1. Preparation of Black Ginseng

*Panax ginseng* was harvested to produce black ginseng from Eumseong-gun, Chungbuk Province, Korea. *P. ginseng* was cultivated in accordance with the protocol of the “Ginseng GAP Standard Cultivation Guide” developed by the Rural Development Administration (RDA), Republic of Korea. The voucher specimen (NIHHS1901) was deposited in the herbarium of the Department of Herbal Crop Research, National Institute of Horticultural and Herbal Science (NIHHS), RDA, Republic of Korea. Roots of five-year-old ginseng were processed into black ginseng in this study. The roots of ginseng were peeled, washed, and dried by hot air and sunlight. Dried roots were steamed at 95–98 °C for 3–5 h and dried at 50 °C for 24 h, and this was repeated three times. Produced black ginseng was dried and homogenized. Powdered sample was extracted twice by reflux extraction with water at 80 °C for 4 h, filtered using a filter paper, and concentrated to 10.8 brix by a vacuum evaporator (Eyela, Tokyo, Japan). For animal administration, 200 mg tablets were produced using the extract of black ginseng.

### 4.2. Animal Adminstration and Sample Collection

The Institutional Animal Care and Use Committee of Chungbuk National University approved all animal experiments (approval no. CBNUA-1218-18-01). Beagle dogs were kept in each cage. Each dog was fed 250 g of standard laboratory diet (Cargill Agri Purina Inc., Sungnam, Korea) with chemical composition as follow: crude protein 25.00%, crude fat 9.00%, crude fiber 4.00%, crude ash 10.00%, calcium 1.00%, phosphorus 1.20%.

Dogs aged from 2 to 3 years old were used, and their body weight range was 8–12 kg. In this study, four healthy beagle dogs (2 females and 2 males) were fed with two tablets of black ginseng (400 mg/10 kg/day) daily for 8 weeks. Blood was collected using BD Vacutainer^®^ SST—II Advance (BD Biosciences, San Jose, CA, USA) after 0, 4, and 8 weeks. The blood samples were centrifuged at 3000 rpm at 4 °C for 15 min to obtain serum.

### 4.3. HR-MAS NMR Measurement

For high-resolution magic angle spinning (HR-MAS) NMR analysis, 36 μL of each sample was mixed with 4 μL of 0.2 M sodium phosphate buffer (pH 7.5) made by deuterium oxide. All samples were measured using a 600.167 MHz Agilent NMR spectrometer, which was equipped with a 4 mm gHX NanoProbe (Agilent Technologies, Santa Clara, CA, USA). All measurements were performed by magic angle spinning at 2000 Hz. The Carr–Purcell–Meiboom–Gill (CPMG) preset pulse sequence was used as a pulse sequence of one-dimensional (1D) NMR spectroscopy for the suppression of high molecular mass compounds and water [43]. In order to find a 90° pulse-width (pw90) for maximizing the signals, an array experiment was performed. The arrayed spectra showed a sine wave with null points (180° and 360°), therefore, it was easy to find a 360° pulse width (pw360). The pw360 was determined to be 34.6 μs, and the pw90 was calculated to 8.65 μs by dividing the pw360 by 4. Big tau, which is the total time for T_2_ relaxation, was set to 0.15 s to filter macromolecule signals such as plasma protein and lipids. The acquisition time was 3.000 s, the relaxation delay was 3.000 s, and 128 total transients were collected.

### 4.4. Data Analysis

Phase and baseline of obtained NMR spectra were corrected manually. Metabolic identification and quantification for each sample were performed using Chenomx NMR Suite 8.4 Professional (Chenomx Inc., Edmonton, AB, Canada) and previously reported literature.

Quantified metabolites were calculated to relative concentrations of the total area. The relative concentrations of metabolites were statistically calculated using MetaboAnalyst 4.0 (https://www.metaboanalyst.ca). Each group was compared with 0 week after feeding of black ginseng to perform the single group t-test. For the multivariate statistical analyses of NMR spectra, the binning process was conducted using Chenomx NMR Suite 8.4 Professional. The binning area of each spectrum was from 0.5 to 8.6 ppm and the binning size was 0.001 ppm. Water, ethanol, and spinning sideband peaks were excluded in the binning area. The binning results were normalized to total area and aligned using the icoshift algorithm of MATLAB R2013b (MathWorks, Natick, MA, USA). Multivariate statistical analyses of processed NMR spectra were conducted using SIMCA 15.0.2 software (Umetrics, Umeå, Sweden). PCA and PLS-DA were analyzed.

## 5. Conclusions

To investigate the efficacy of black ginseng, which has significant biological activity, beagle dogs were fed black ginseng for eight weeks, and small blood samples were analyzed with HR-MAS NMR without further pretreatment. Differences over the period of feeding of black ginseng were also evident in multivariate statistical analysis, and statistical analyses of metabolite profiles also identified changes in metabolites that could indicate health improvements such as increased immunity and increased energy metabolism. The biological effects of black ginseng were confirmed through metabolomics analysis, and furthermore, it was confirmed that metabolomics can be applied to the pet healthcare industry.

## Figures and Tables

**Table 1:** Identified and quantified metabolites in 600 MHz HR-MAS NMR spectra from dog serum. Relative concentrations were calculated against the total area.

Compounds	Chemical Shifts (Multiplicities) (ppm)	0 Week	4 Weeks	8 Weeks
		(Mean (%) ± Standard Deviation)		
Acetate	1.93 (s)	0.211 ± 0.064	0.244 ± 0.066	0.189 ± 0.062
Alanine	1.49 (d), 3.79 (q)	5.180 ± 0.946	7.031 ± 1.095 ^a^	6.531 ± 2.079
Citrate	2.54 (d), 2.69 (d)	0.878 ± 0.644	0.576 ± 0.126	0.734 ± 0.322
Creatine	3.05 (s), 3.94 (s)	0.403 ± 0.199	0.411 ± 0.181	0.374 ± 0.166
Formate	8.46 (s)	0.660 ± 0.109	0.430 ± 0.098 ^a^	0.490 ± 0.102 ^a^
Glucose	3.26 (dd), 3.40–3.45 (m), 3.47–3.54 (m), 3.55 (dd), 3.76–3.80 (m), 3.86–3.91 (m), 4.66 (d), 5.23 (d)	41.627 ± 4.529	42.265 ± 7.364	44.514 ± 4.092
Glutamate	2.13–2.05 (m), 2.33–2.36 (m)	0.839 ± 0.295	1.000 ± 0.170	0.936 ± 0.139
Glutamine	2.12–2.15 (m), 2.44–2.48 (m), 3.77 (t)	6.494 ± 0.155	7.483 ± 0.856	8.851 ± 1.061 ^b^
Glycerol	3.57 (dd), 3.67 (dd), 3.76 (m)	0.734 ± 0.104	0.649 ± 0.078	0.568 ± 0.164
Glycine	3.57 (s)	2.800 ± 0.457	3.011 ± 0.280	2.947 ± 0.619
Histidine	7.06(s), 7.77 (s)	1.073 ± 0.187	1.329 ± 0.327	1.431 ± 0.099 ^b^
Isoleucine	0.95 (t), 1.02 (d), 1.25 (m), 1.46 (m), 1.97 (m), 3.67 (d)	0.415 ± 0.150	0.572 ± 0.099	0.530 ± 0.105
Lactate	1.34 (d), 4.16 (q)	25.640 ± 8.500	24.010 ± 8.663	20.714 ± 9.561
Leucine	0.97 (t), 1.68–1.74 (m), 3.73 (m)	1.032 ± 0.204	1.284 ± 0.274	1.328 ± 0.326
Methionine	2.15 (s), 2.16 (m), 2.65 (t)	0.531 ± 0.097	0.461 ± 0.064	0.470 ± 0.066
Proline	1.99–2.07 (m), 2.36 (m), 3.35 (q), 3.43 (q), 4.14 (dd)	3.325 ± 0.915	2.247 ± 0.330	2.425 ± 0.412
Pyruvate	2.38 (s)	0.758 ± 0.252	0.518 ± 0.116	0.409 ± 0.110
Serine	3.85 (dd), 3.99 (s)	2.892 ± 0.690	2.129 ± 0.336	2.366 ± 0.469
Succinate	2.41 (s)	0.085 ± 0.010	0.075 ± 0.001	0.072 ± 0.007
Threonine	1.33 (d), 3.58 (d), 4.26 (m)	3.364 ± 1.148	2.850 ± 0.556	2.663 ± 0.668
Valine	1.00 (d), 1.05 (d), 2.27 (m), 3.62 (d)	1.060 ± 0.260	1.444 ± 0.323 ^a^	1.458 ± 0.409 ^a^

^a^ Statistically different from 0 week with *p*-value < 0.05; ^b^ Statistically different from 0 week with *p*-value < 0.01.

**Table 2:** Area under the curve (AUC) values of metabolites over 0.7 obtained from biomarker analysis of groups of before and after feeding of black ginseng.

Name	AUC	*p*-Value	Log_2_FC	Name	AUC	*p*-Value	Log_2_FC
Valine	0.969	0.005	−0.255	Serine	0.844	0.066	0.287
Formate	0.938	0.009	0.155	Alanine	0.813	0.041	−0.357
Glutamine	0.938	0.008	−0.310	Glutamate	0.813	0.112	−0.097
Histidine	0.906	0.025	−0.204	Glycine	0.797	0.136	−0.076
Isoleucine	0.906	0.021	−0.107	Glycerol	0.781	0.216	0.096
Leucine	0.906	0.028	−0.182	Pyruvate	0.781	0.047	0.223
Proline	0.906	0.033	0.407	Methionine	0.750	0.107	0.051
				Glucose	0.719	0.299	−0.056
